# Endogenous and artificial miRNAs explore a rich variety of conformations: a potential relationship between secondary structure and biological functionality

**DOI:** 10.1038/s41598-019-57289-8

**Published:** 2020-01-16

**Authors:** C. M. A. Gangemi, S. Alaimo, A. Pulvirenti, Sara García-Viñuales, D. Milardi, A. P. Falanga, M. E. Fragalà, G. Oliviero, G. Piccialli, N. Borbone, A. Ferro, A. D’Urso, C. M. Croce, R. Purrello

**Affiliations:** 1 Department of Chemical Science, University of Catania, Viale A. Doria 6, 95125 Catania, Italy; 2 Bioinformatics Unit, Department of Clinical and Experimental Medicine, University of Catania, Italy c/o Department of Mathematics and Computer Science, Viale A. Doria 6, 95125 Catania, Italy; 3 Istituto di Cristallografia CNR, Via P. Gaifami 9, 95126 Catania, Italy; 4 Department of Molecular Medicine and Medical Biotechnologies, University of Naples Federico II, Via Pansini 5, 80131 Napoli, Italy; 5 Department of Pharmacy, University of Naples Federico II, D. Montesano 49, 80131 Napoli, Italy; 6 Department of Molecular Virology, Immunology and Medical Genetics, Comprehensive Cancer Center, The Ohio State University, Columbus, OH 43210 USA

**Keywords:** RNA, RNA

## Abstract

Mature microRNAs are short non-coding RNA sequences which upon incorporation into the RISC ribonucleoprotein complex, play a crucial role in regulation of gene expression. However, miRNAs can exist within the cell also as free molecules fulfilling their biological activity. Therefore, it is emerging that in addition to sequence even the structure adopted by mature miRNAs might play an important role to reach the target. Indeed, we analysed by several spectroscopic techniques the secondary structures of two artificial miRNAs selected by computational tool (miR-Synth) as best candidates to silence c-MET and EGFR genes and of two endogenous miRNAs (miR-15a and miR-15b) having the same seed region, but different biological activity. Our results demonstrate that both endogenous and artificial miRNAs can arrange in several 3D-structures which affect their activity and selectivity toward the targets.

## Introduction

MicroRNAs (miRNAs) are a class of highly conserved, short (18–24 nucleotides) and non-coding single-stranded RNA, with a crucial role in different cellular and metabolic pathways^1–4^. They act as key post-transcriptional regulators of gene expression in development^3,5^, immunity^6^ and peptide bond formation^7^. The normal expression of miRNAs is important in physiological processes, while the aberrant expression of miRNAs is often associated to the initiation and development of human diseases like cancer, genetic disorders and altered immune system functions^1,8,9^. Since the early 2000s, a systematic profiling of several human cancer samples showed that changes in miRNA concentration, as consequence of up- or down-regulation in their biogenesis, are correlated with the development and differentiation of cancer cells, thus providing good diagnostic biomarkers for a great variety of cancers^10–12^. The discovery of cancer-promoting miRNAs has been accompanied also by the identification of many cancer-suppressing miRNAs, such as miR-15a and miR-16-1, which are able to inhibit tumorigenesis driven by the Bcl2 oncogene^13–19^.

So far, over 2000 miRNAs have been identified in humans, and they target most of human protein coding genes. Mature miRNAs are obtained through a series of steps starting from 1–3 Kb long RNA precursors called pri-miRNAs^4^. These are transcribed in the nucleus by RNA polymerase II and processed by the RNase III enzyme Drosha and the double-stranded RNA-binding protein (dsRNAbd) to produce hairpin-shaped secondary precursors called pre-miRNAs (~60–100 nucleotides)^20–22^. Next, pre-miRNAs are transported by Exportin-5 to the cytoplasm where they are further cleaved by the RNase III Dicer to give the mature double stranded miRNAs (~18–24 nucleobases)^23,24^. Following the separation of the two strands, the strand known as “guide strand” is then incorporated into the RISC ribonucleoprotein complex (miRNA-Induced Silencing Complex) which binds and silences the complementary target mRNA, whereas the other strand, known as the “passenger strand”, is degraded or involved in the regulation of miRNA homeostasis^25^. In the RISC complex, the mature miRNA can perfectly or imperfectly match with the complementary mRNA inducing its degradation or inhibition^26^. Usually, in plants this matching is nearly perfect, and the mechanism involves the RNA interference machinery^27^. On the contrary, in animal cells mRNAs are imperfectly matched by miRNAs and the mechanism, not well known, seems to preserve the mRNA^28^. In particular, in mammalians it has been supposed that the specificity is not restricted to the seed region, but it has been reported that the stabilization of miRNA-mRNA double strand can involve also the residues in position 13–16 from the 5′ end of miRNA, especially in the case of imperfect matching^29^. Furthermore, it is known that a single mature miRNA can control the expression of thousands target mRNAs and a single mRNA is targeted by multiple miRNAs. Previously, it has been reported that the miRNAs binding availability to target mRNAs is highly dependent on mRNAs structure^30^. This means that the base-pairing efficiency cannot be considered the only parameter which affects the functionality of miRNAs regulation. Only recently, it is emerging that even the structure of mature miRNAs might play an important role^31–34^. Thus, potential arrangements of miRNAs on 3D structures can act as an additional tuning level of post-transcriptional regulation, influencing the affinity and the specificity for their targets. Therefore, nowadays, miRNAs are emerging as novel biological targets for chemists, biochemists and bioinformatics to design synthetic miRNAs able to target multiple genes and consequently to find therapies for diseases caused by miRNAs dysregulation^35^.

Very recently, a team of biologists and bioinformatics leaded by Croce and Ferro, respectively, have exploited the ability of endogenous miRNAs to target multiple sites of genes. The goal was to develop the bioinformatics tool “miR-Synth” aimed at designing artificial microRNAs (a-miR) capable to target multiple genes in multiple sites^36^. A scoring function ranked the designed miRNAs according the predicted repression efficiencies, and the system was validated testing the silencing efficiency of single-stranded and double-stranded miRNAs against c-MET and EGFR, two genes associated with lung cancer. However, among the top-six ranked miRNAs displayed by “miR-Synth”, two of them (a-miR-23 and a-miR-98) did not show significant inhibition of the expression of c-MET and EGFR, indicating that these artificial miRNAs do not elicit efficient anticancer activity^36^. Though, “miR-Synth” tool does not consider the role of possible secondary structures adopted by the miRNA sequences, which has been recently evaluated as an important factor likely affecting the miRNAs function^31–34^. On the basis of this hypothesis, we decided to investigate by several techniques, such as electronic circular dichroism (ECD), differential scanning calorimetry (DSC), nuclear magnetic resonance (^1^H-NMR) and non-denaturing polyacrylamide gel electrophoresis (PAGE), the existence of secondary structures which could be correlated to the activity of these sequences. To confirm our hypothesis, we performed a structural investigation on two top-ranked artificial miRNAs, which induced a significant inhibition of the luciferase activity for both c-MET and EGFR (a-miR-141 and a-miR-196), and as comparison we investigated the structure of two a-miRNAs which seemed to be inactive (a-miR-23 and a-miR-98). Finally, we carried out similar experiments with two endogenous miRNA sequences (mir-15a and mir-15b) having the same seed sequence but distinct biological function^10^, in order to demonstrate the important role played by the adopted structure. As a result the prediction efficiency of computational resource (as “miR-Synth”) will be definitively improved, considering the potentiality of miRNA sequences to adopt several secondary structures as new constrain to design efficient miRNA sequences.

## Results and Discussion

**Figure 1:**
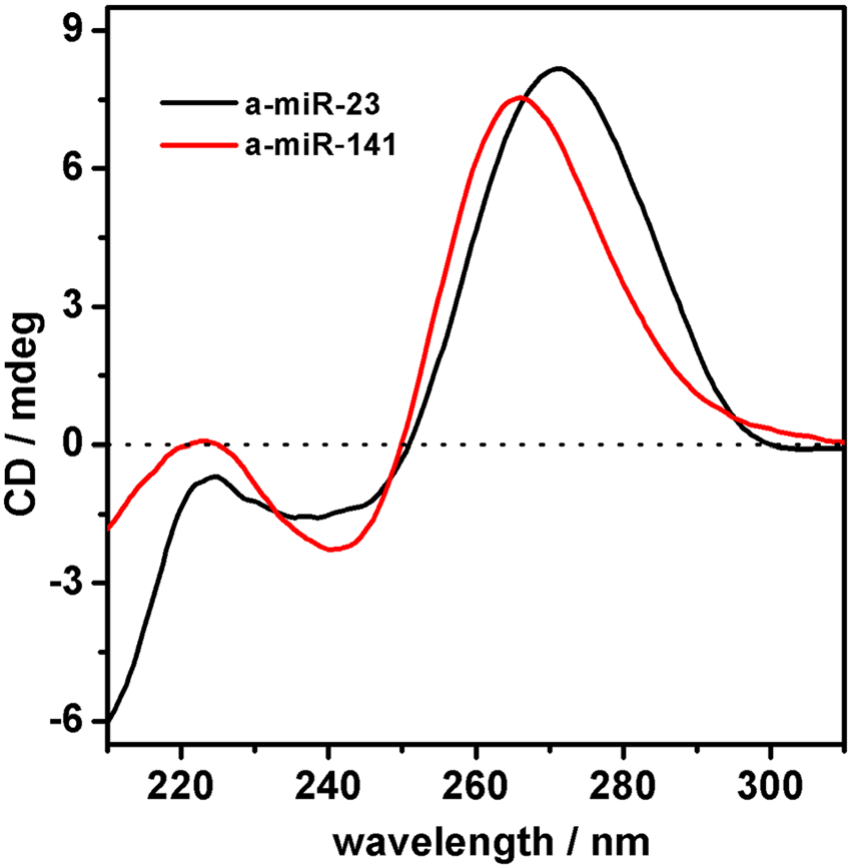
CD spectra. CD-spectra of 1.4 μM a-miR-23 (black curve) and a-miR-141 (red curve) in PBS 10 mM, pH = 6.8, 37 °C.

First of all, we performed ECD measurements and melting experiments of the four artificial miRNA sequences (Figs. 1 and S1). ECD spectroscopy is a very powerful diagnostic tool for monitoring the optical activity that generates, within a specific electronic transition, by the absence of roto-reflection symmetry elements in the chromophore responsible for the transition. Noteworthy, both the intensity and the shape of the ECD signals are very indicative for the occurrence of electronic coupling between chromophores^37^. In the case of single nucleotides the low intensity ECD signal arises from the presence of the chiral sugar that “donates” chirality to the whole molecule. Nucleotides linked by phosphodiester bond can assume various arrangements (e.g. helix); the reciprocal interactions of transition dipole moments of the bases changes with the structure and modulates both the shape and the intensity of the signal becoming highly diagnostic of the specific structure. For example, self-structured single filaments are characterized by ECD signal more intense than that observed in the case of isolated nucleotides or unstructured sequences with random conformations. Noteworthy, the ECD intensity is further enhanced in the case of double-stranded helix: the number of base pairs per turn, the inclination and the distance of bases with respect to the helix axis, the rise per base pair and the handedness of the helix, contribute to the ECD signal, explaining why this spectroscopic technique has been used extensively to study the conformational properties of nucleic acid constructs^38^.

In contrast to what expected for single strands with a large number of random conformations (i.e.; intensity of the signal close to few millidegrees and mono signated bands), the ECD spectra of all the a-miRs tested (a-miR-23, a-miR-141, a-miR-98and a-miR-196) show an intense positive band centred at ~270 nm and a less intense negative band centred near 240 nm, suggesting that the studied a-miRs are not in random conformations. Indeed, the ECD spectra in Fig. 1 correspond to those of A-form RNA of heterogeneous primary structures, indicating that probably the studied sequences are folded in ordered secondary structures (e.g. self-dimers or hairpins). Yet, although the shape of the ECD signals for all the a-miRs studied seems quite similar, the differences observed between them do suggest that each sequence might adopt distinct secondary structures.

**Figure 2:**
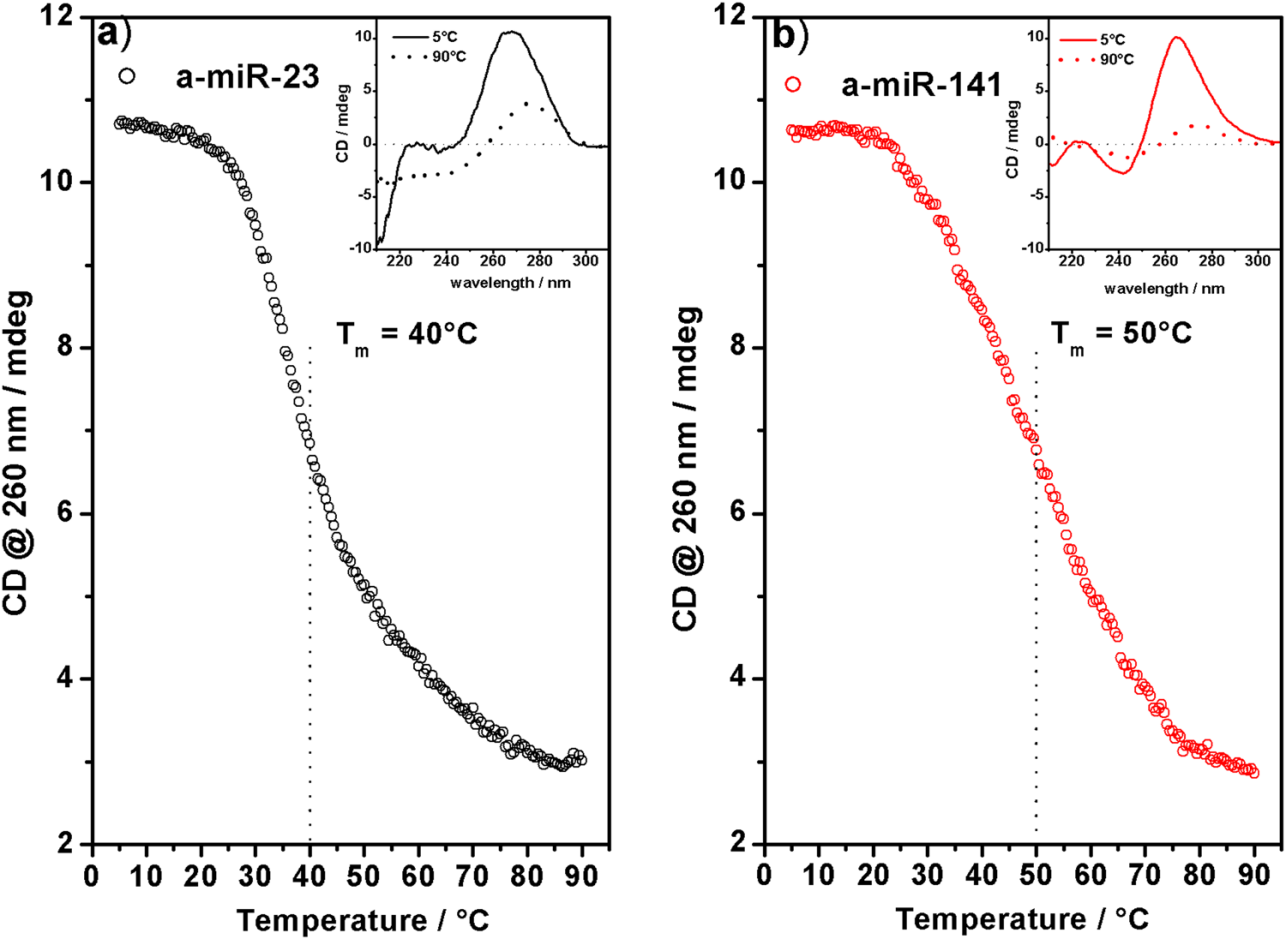
CD melting. CD-melting curves of (**a**) a-miR-23 and (**b**) a-miR-98 in PBS 10 mM, pH = 6.8. Insets show the CD spectra of a-miR sequences at 5 °C (solid curves) and 90 °C (dotted curves).

In order to verify this hypothesis, we performed melting experiments by monitoring the variation of the ECD signal at temperatures in the range 5–90 °C. Before presenting the experimental results, it is worth describing the change expected with temperature in the ECD signal for solutions containing nucleic acid sequences in which: (i) the nucleobases are not involved in hydrogen bonds or (ii) the nucleobases forms “internal” (e.g. hairpins) or external (e.g. duplexes) base-pairing, respectively. In the first case, the increase of the temperature causes “only” the breaking of π-π interactions (in the case of highly structured single strands). As a consequence, the ECD intensity will decrease linearly upon increasing the temperature together with the smooth loss of base stacking. On the contrary, breaking of hydrogen bonds and subsequent “melting” of the base-base coupling is accompanied by a net transition evidenced by sharp decrease of the ECD intensity, which is centred at the temperature where 50% of denaturation occurs (T_m_). In general, the loss of ordered structure by heating causes ECD changes which, monitored at a fixed wavelength as a function of temperature, allow to estimate the stability of secondary structures. The melting experiments show a clear transition for a-miR-23, -141 (Fig. 2) and -196 (Fig. S2), but not for a-miR-98 (Fig. S2), whose ECD decreases smoothly with temperature. In particular, the CD-melting curves of a-miR-23, -141 and -196 sequences show one evident melting event in the interval 40–50 °C, suggesting that these a-miRs adopt well-defined folded structures. The melting of the a-miR-196 sequence shows an additional melting event at ~20 °C.

In order to better evaluate a possible correlation between the structure and the biological efficiency, we decided to perform further studies by DSC, ^1^H-NMR and 2D-PAGE experiments on the first two sequences of the top-six ranked miRNAs displayed by “miR-Synth” (see Table S1 of ref. ^36^): one active in silencing c-MET and EGFR genes (a-miR-141) and one inactive for the same test (a-miR-23).

**Figure 3:**
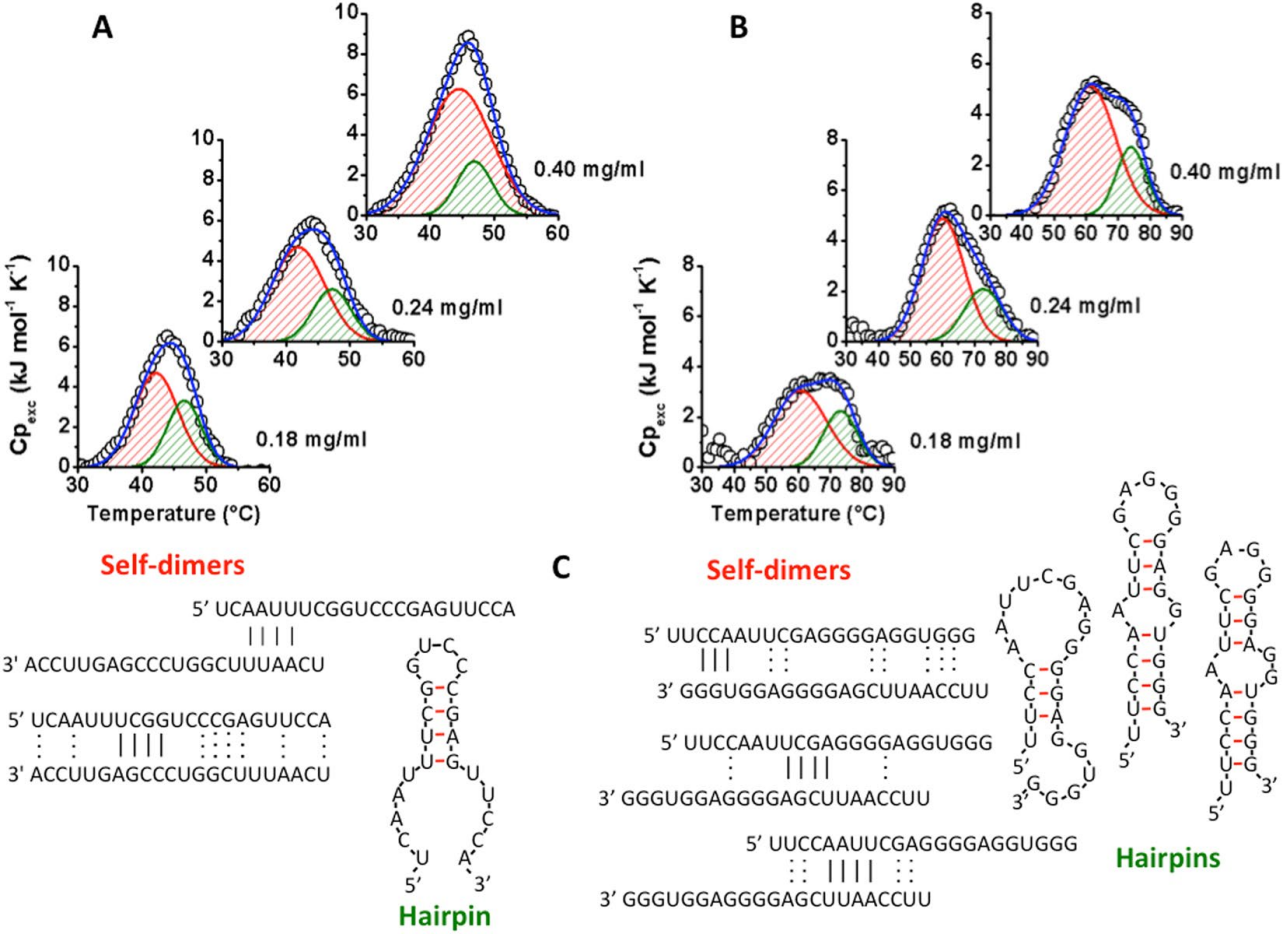
DSC measurements. DSC curves of a-miR-23 (**A**) and a-miR-141 (**B**) of unfolding dependence on strand concentration (0.18, 0.24 and 0.40 mg/mL) in 10 mM PBS, 100 mM NaCl at pH 6.8. DSC curves (black open circles) are deconvoluted in two components: the red and green curves correspond to the 1^st^ and 2^nd^ transition of a biphasic melting curve. The sum of the two components is reported as a blue line. (**C**) Cartoon representations of possible structures (self-dimers and hairpins) of a-miR-23 and a-miR-141, calculated by “IDT^®^ oligoanalyzer tool”.

Differential Scanning Calorimetry (DSC) was used to measure the temperature and the enthalpy changes associated to unfolding/dissociation of RNA strands. Noteworthy, DSC analysis allowed us to explore temperature-induced transitions in a concentration range (0.18–0.40 mg/mL) to figure out the unfolding parameters dependence on sample concentration in order to establish the molecularity of thermal transition. Indeed, monomolecular transitions (e.g. those associated to unfolding of hairpins) are virtually independent on strand concentration, while transitions with higher molecularities, as those associated to dissociation of self-dimers, will exhibit a significant dependence on sample concentration. Figure 3A reports the Cp_exc_(T) traces of a-miR-23 at different strand concentrations. It is evident that the a-miR-23 DSC trace has a complex shape that can be fitted using at least two components (Figs. 3 and S3). The temperatures (T_m1_) and enthalpy changes (ΔH_1_) associated to the first component exhibit a concentration-dependent behavior: indeed, T_m1_ and ΔH_1_ shifts from 42.1 up to 44.5 °C and from 40 kJ/mol up to 80 kJ/mol, respectively as concentration increases (Table S1). By contrast, the unfolding parameters of the second peak (T_m2_ ~ 47 °C and ΔH_2_ ~ 20 kJ/mol) are constant over the whole explored range of concentrations and in accordance to UV-CD melting curves. These results indicate that the first component is mainly ascribable to dissociation of self-dimers, while the second one may be attributed to monomolecular events i.e. unfolding of hairpins. A comparative analysis of DSC traces of rescanned samples (Fig. S4) supports this hypothesis: with the exception of samples at 0.40 mg/mL where irreversible phenomena are more likely favored, the first component of the re-heating traces always increases their enthalpies at the expense of the second component. This means that after the first heating, unfolded hairpins may associate thus contributing to increase the population of self-dimers.

Similar to a-miR-23, the compound a-miR-141 exhibits a biphasic DSC transition (Fig. 3B, Table S1 and Fig. S5). The first component is centered at about T_m1_ ~ 60.5 °C and its position does not vary with concentration. However, the unfolding enthalpy ΔH_1_ is concentration-dependent ranging from about 63 kJ/mol (C = 0.18 mg/mL) to 101 kJ/mol (C = 0.40 mg/mL). Conversely, the second component is concentration independent (T_m2_ ~ 73 °C and ΔH_2_ ~ 30 kJ/mol). Next, a comparative analysis with a second heating of this RNA sequence evidences a significant decrease of the melting temperatures of both peaks coupled with a decrease in the transition enthalpies (Table S1 and Fig. S6). On the whole, these data point to a very complex thermal profile for the a-miR-141 sequence: the first component is more likely ascribable to the dissociation of self-dimers while the second one is a monomolecular event ascribable to the unfolding of monomolecular hairpins. However, the relative amounts of different types of hairpins or self-dimers appear modified in a second heating run which causes a redistribution in the populations of the different structures.

**Figure 4:**
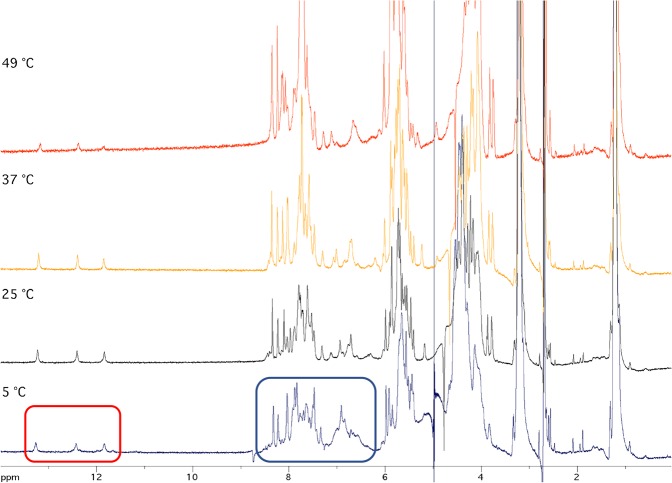
^1^H-NMR spectra.^1^H-NMR spectra of a-miR-23 in PBS recorded at different temperatures. The blue box highlights the anomeric and aromatic protons. The red box highlights the H-bonded imino protons.

At the light of ECD and DSC results, suggesting the different arrangements adopted by a-miR-23 and a-miR-141, we performed ^1^H-NMR analyses in order to clarify this aspect. The water-suppressed ^1^H-NMR spectra of a-miR-23 recorded in PBS at increasing temperatures were in agreement with the presence in solution of a single intramolecular secondary structure (Fig. 4).

In particular, the observation of three sharp and well resolved signals at 11.8–13.2 ppm, attributable to three exchange-protected H-bonded imino protons of guanosines involved in G-C or G-U base pairs, suggested that a-miR-23 might fold into an intramolecular hairpin. Furthermore, the comparison of the relative intensity of the imino proton signals at the different temperatures suggested that the melting temperature of the hypothesized hairpin structure is higher than 37 °C and lower than 49 °C, in agreement with the CD melting and DSC evidences.

Conversely, the ^1^H-NMR spectra of a-miR-141 did not show the presence of a single well-defined secondary structure (Fig. S7). In fact, the NMR data do not show any sharp downfield-shifted signal attributable to exchange-protected N1H or N3H proton of guanosines or uridines, respectively, involved in Watson-Crick or in other non-canonical hydrogen-bonding schemes. Nonetheless, the presence of broad envelope peaks between 5.5 and 8.5 ppm instead of discrete peaks belonging to anomeric and aromatic protons, respectively, suggested that the a-miR-141 RNA strand folds into several secondary structures whose rate of interconversion is faster than the NMR timescale.

**Figure 5:**
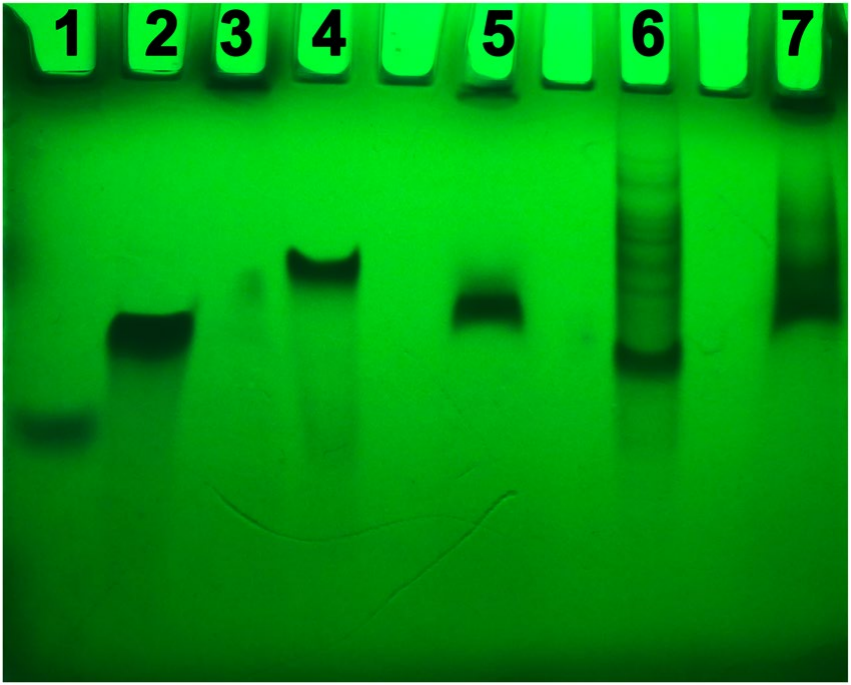
PAGE experiments. PAGE of a-miR-23 and a-miR-141 performed 72 h after incubation at 37 °C with DMEM buffer (lanes 4 and 6, respectively) or DMEM + 10% FBS (lanes 5 and 7, respectively). The PAGE behaviour of the control T_24_ RNA sequence in the absence or presence of FBS nucleases is shown in lanes 2 and 3, respectively. All samples were loaded at 175 µM concentration in the same run. The running marker bromophenol blue was loaded in lane 1.

Once analysed the structural features of artificial miRNAs, we tested the resistance of both a-miR-23 and a-miR-141 from being digested by nucleases found in the fetal bovine serum (FBS). Usually, the formation of ordered secondary structures by suitable nucleic acids protects them from being digested by nucleases found in the FBS, thus allowing them to reach the target. Therefore, considering that the electrophoretic mobility is a function of the length, conformation and charge of the molecules, we compared the PAGE experiments performed on both a-miR-23 and a-miR-141 before and after incubation for 72 h with FBS at 37 °C (Fig. 5). As a result, following its incubation with FBS (lane 7), a-miR-141 did not show any degradation product compared to the same sequence without FBS treatment (lane 6). Conversely, in the case of a-miR-23 the incubation with FBS led to the appearance of a single band (lane 5), slightly faster than control (lane 4), indicating that a partial degradation occurred.

In summary, considering the large melting curve with higher T_m_ and the broadened NMR signals of a-miR-141, it is plausible to hypothesize that this sequence exists at 37 °C as a mixture of well-organized secondary structures which protect it from being digested by FBS proteases. Whereas the sole secondary structure formed by a-miR-23 is not as stable to withstand the activity of FBS ribonucleases; indeed, at 37 °C it starts to melt. Therefore, the different thermal and enzymatic stability of the secondary structures adopted by the two sequences may explain the results of the biological assay obtained in the previous work^36^, which indicated that the sequence adopting stable conformations reaches the targets and performs its biological function.

**Figure 6:**
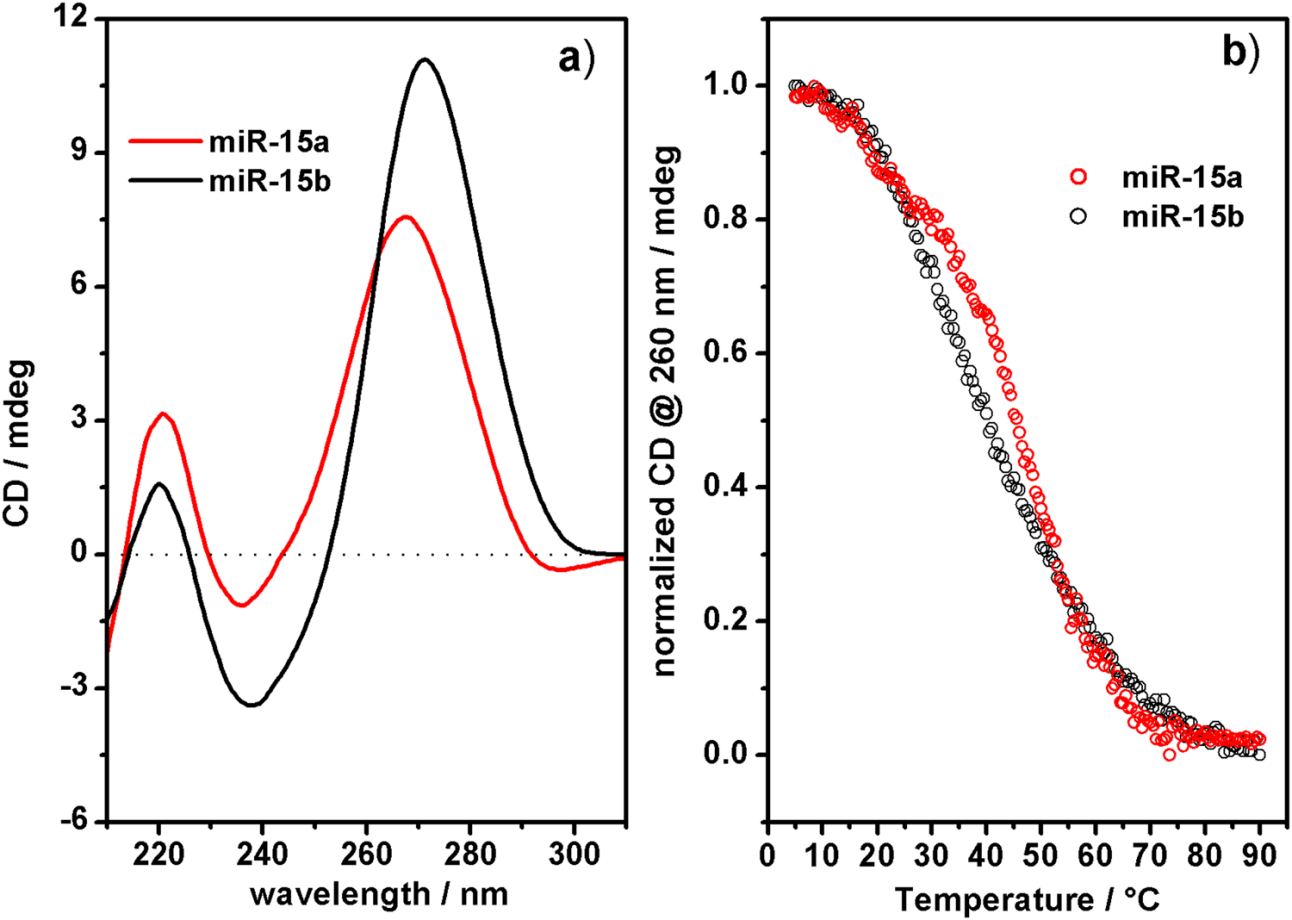
CD and melting experiments. CD spectra (**a**) and CD-Melting curves at 260 nm (**b**) of miR-15a (red curve) and miR-15b (black curve), 2.8 μM in PBS 10 mM at pH = 6.8 at 37 °C.

Intrigued by the apparent correlation between the different biological behaviour and the structural properties of a-miR-23 and a-miR-141, we decided to investigate the structural properties of two endogenous human miRNA 22-meric sequences, miR-15a and miR-15b, which differ only by four out of twenty-two nucleotides (see Supporting Information). In particular, miR-15b differs from miR-15a for the replacement of the A12 purine (pu) in the middle of the sequence with the pyrimidine (py) C12, and for the replacement of the GUG (pu-py-pu) sequence at the 3′-end with the alternative pu-py-pu 3-mer ACA. Noteworthy, although miR-15a and miR-15b present the same seed sequence, they are involved in distinct biological functionalities, which are related to the pathogenesis of chronic lymphocytic leukaemia for miR-15a^16^ and progression of metastasis for miR-15b^10,39^. We speculate that different secondary structures adopted by the two miRNA sequences might be correlated to the distinct biological functions and affect the affinity toward the target. The ECD spectra of miR-15a and miR-15b recorded in PBS at 37 °C show an electronic coupling between the bases for both sequences, more intense for miR-15b, confirming that both endogenous miRNAs fold into one or more ordered secondary structures in PBS at room temperature (Fig. 6a).

Although the similarity in the ECD profiles of miR-15a and miR-15b, characterized by a minimum at around 235 nm and a maximum at around 265 nm, suggests the formation of similar secondary structures, the comparison of their normalized CD melting curves indicates a different thermal behaviour of the secondary structures formed by miR-15a or miR-15b (Fig. 6b). We observed two different melting events for miR-15a, the first at ~20 °C and the second at ~45 °C, indicating the presence of at least two different folded structures; whereas a single melting event at ~40 °C accounted for the presence of a single folded species in the case of miR-15b. To obtain further structural insights on miR-15a and miR-15b, we performed DSC and ^1^H-NMR experiments.

**Figure 7:**
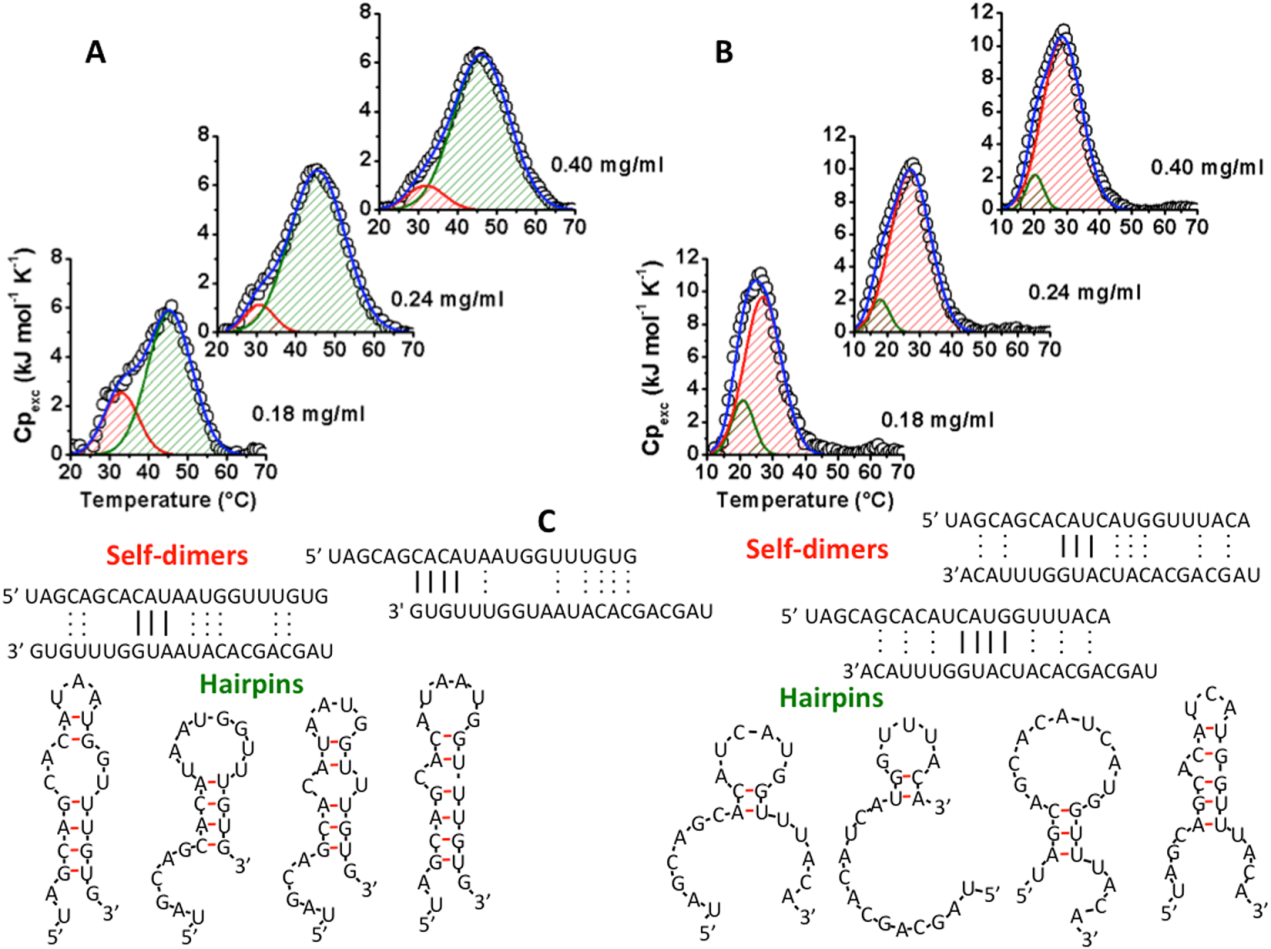
DSC measurements. DSC curves of miR-15-a (**A**) and miR-15-b (**B**) of unfolding dependence on strand concentration (0.18, 0.24 and 0.40 mg/mL) in 10 mM PBS, 100 mM NaCl at pH 6.8. DSC curves (black open circles) are deconvoluted in two components: the red and green curves correspond to the 1^st^ and 2^nd^ transitionof a biphasic melting curve. The sum of the two components is reported as a blue line. (**C**) Cartoon representations of possible structures (self-dimers and hairpins) of miR-15-a and miR-15-b calculated by “IDT^®^ oligoanalyzer tool”.

DSC traces of miR-15a evidenced a large endothermal peak centred at about 45.5 °C with a small shoulder located at about 31.5 °C (Fig. 7A, Table S2 and Fig. S8). Although the melting temperatures of both transitions do not depend on concentration, the ΔH values significantly increase as sample concentrations rise (Table S2). Altogether these results demonstrate that the unfolding of monomolecular hairpins occurs at a temperature of about 45 °C. However, self-dimers dissociation events are convoluted within the large peak at 45 °C. Other phenomena, likely ascribable to dissociation of different populations of self-dimers, occur also at lower temperatures (T ~ 32 °C). Moreover, DSC traces relative to re-heating runs (Fig. S9) evidenced only a single, concentration-independent peak centred at a temperature of about 46 °C and with an enthalpy change ΔH ranging from 43.7 to 48.8 kJ/mol ascribable to hairpin unfolding, suggesting that the dissociation of self-dimers is an irreversible phenomenon.

The thermal transitions of miR-15b are centred at lower temperatures than miR-15a (Fig. 7B, Table S2, Figure S10). In particular, DSC traces of miR-15b may be deconvoluted in two components located at T_m1_ ~ 20 °C and T_m2_ ~ 27 °C. Although melting temperatures do not depend on strand concentration, the enthalpy changes associated to the 1^st^ transition decrease at increasing strand concentration. Conversely, enthalpies of the 2^nd^ component increase as concentration rises and counterbalance the corresponding decrease of the preceding transition; as a result, the sum of the enthalpies of the two components (ΔH_1_ + ΔH_2_) is constant and independent on strand concentration. These data suggest that the first transition may be mainly ascribed to monomolecular hairpin unfolding events and the second one to self-dimers dissociation. Both these events are highly reversible because their thermal behaviour is unchanged in a second DSC heating scan (Fig. S11). Noteworthy, DSC data highlighted the different stability of the structures adopted by the two endogenous miRNAs.

**Figure 8:**
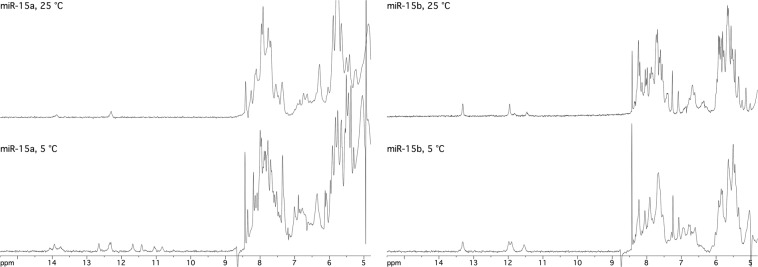
^1^H-NMR. Downfield region of water-suppressed 1H-NMR spectra of miR-15a (left) and miR-15b (right) dissolved at 0.3 mM in 10 mM PBS, pH 7.

The ^1^H NMR spectra of miR-15a and miR-15b (Fig. 8) confirmed the formation of ordered secondary structures for both the selected endogenous miRNAs, as disclosed by the observation of several signals belonging to exchange-protected H-bonded imino protons in the water suppressed NMR spectra recorded at 5 °C. Differently for what we found for a-miR-141 and a-miR-23, the apparent melting temperatures suggested by the NMR spectra recorded at increasing temperature were significantly lower than those calculated on the basis of ECD and DSC evidence. Indeed, at physiological temperature we did not observe any imino proton signal for both endogenous miRNAs and already at 25 °C most of those signals were not detectable. However, the number of imino proton signals observed for the two endogenous miRNAs at 5 °C was in agreement with the indications obtained by the above mentioned CD melting evidence and indicative for the formation of more than one secondary structure for miR-15a and of a single secondary structure for miR-15b (Fig. 8).

This latter indication was also confirmed by the nucleases resistance PAGE assay performed on miR-15a and miR-15 (Fig. S12), which at the same time confirmed the resistance of both endogenous miRNAs toward the endo- and exo-nucleases present in the FBS (we did not observe any faster migrating degradation product in the samples incubated with FBS), as well as the presence of more than one secondary structure for both miRNAs especially in the biological environment simulated by the presence of FBS.

Taken together, all data obtained by several techniques converge toward the idea that although the two endogenous miRNAs have very similar sequence and identical seed region, they adopt distinct secondary structures which address them toward different targets.

## Conclusion

The experiments performed on the selected miRNAs have shown that once dissolved in PBS buffer all sequences do not stand in solution as random conformation but, as pointed out by spectroscopic evidence, fold into several 3D-structures which could affect, in particular in a biological environment, their activity and selectivity toward the target. These results suggest that the sequence of natural and synthetic oligonucleotides cannot be considered as the sole parameter to take care of in predicting their biological function. Other constraints have to be considered to describe such complex systems which involve the interaction between miRNAs and enzymes. In these cases, the shape given by supramolecular weak interactions, the number and the stability of accessible structures could play a crucial role not only *in vitro* but mostly *in vivo*. In fact, as demonstrated by the study performed for miR-15a and 15b, differences of few nucleotides, with the replacement of a pyrimidine in place of a purine in the central part of the sequence, deeply changes the folding mode of the mature miRNA. Consequently, their different arrangement could affect the binding with key proteins in the cell, determining the activity of miRNA as post-transcriptional regulation agent. Considering that recently it has been demonstrated that miRNAs can exist within the cell as free molecules owing to the smaller amount of Ago proteins^40–42^, this work highlights the possibility that the miRNAs which are not involved in the RISC complex may exist as ordered secondary structures to fulfil their biological functions.

## Methods

All the artificial miRNAs (a-miRs) and human miRNAs used in our work were purchased from Integrated DNA Technologies IDT^®^ and used without further purification. Each solid was dissolved in ultra-pure water obtained by Elga Purelab Flex system by Veolia with purity of 18.2 MΩcm, achieving stock solutions with concentration of ~100 µM. Then, by dilution in PBS buffer 10 mM ([KCl] 2.7 mM; [NaCl] 137 mM; pH 6.8) we prepared work and sample solutions. Concentration of a-miRs solutions were checked by UV-Vis measurements using the extinction coefficient for each sequence given by IDT: a-miR-23 ε_260 nm_ = 181,600 L/(mol·cm); a-miR-141 ε_260 nm_ = 211,700 L/(mol·cm); a-miR-98 ε_260 nm_ = 194,200 L/(mol·cm); a-miR-196 ε_260 nm_ = 202,200 L/(mol·cm); miR-15a ε_260 nm_ = 209,900 L/(mol·cm); miR-15b ε_260 nm_ = 211,000 L/(mol·cm).

The sequences of miRNAs used in this study are the following (in *italics* we show the seed region and in **bold** the differences between miR-15a and miR-15b):

a-miR-23: 5′-*UCA AUU UCG* GUC CCG AGU UCC A-3′;

a-miR-141: 5′-*UUC CAA UUC* GAG GGG AGG UGG G-3′;

a-miR-98: 5′-*UUU CUU AAG* CAC GCC GUU GGG G-3′;

a-miR-196: 5′-*UGA GUU UCU* CAG CGA CGG ACC G-3′;

miR-15a: 5′-*UAG CAG CAC* AU**A** AUG GUU U**GU G**-3′;

miR-15b: 5′-*UAG CAG CAC* AU**C** AUG GUU U**AC A**-3′

By using the “oligoanalyzer tool” provided by IDT^®^ we calculated the conceivable secondary structures likely adopted by each sequence.

### Electronic circular dichroism and melting experiments

miRNA samples were analyzed at either 1.4 or 2.8 μM single strand concentration. ECD spectra were recorded at 37 °C using a Jasco J-710 spectropolarimeter equipped with a single position Peltier temperature control system. A quartz cuvette with a 1 cm path length was used for all ECD experiments. Conditions were as follows: scanning rate 50 nm/min, data pitch 0.5 nm, digital integration time (D.I.T) 2 s, band width 2.0 nm. Each ECD spectrum was an average of at least five scans. The ECD melting experiments were performed within the temperature range 5–90 °C using a temperature heating rate of 1 °C/min, monitoring the intensity of the miRNA ECD signal at 260 nm.

### Differential scanning calorimetry (DSC)

Differential Scanning Calorimetry (DSC) experiments were carried out using a NanoDSC instrument (TA Instruments). Samples were analyzed at total strand concentrations of  0.18 mg/mL,  0.24 mg/mL and  0.40 mg/mL (i.e. 25 µM, 35 µM and 55 µM, respectively) in a 10 mM PBS buffer pH 6.8. Each sample was heated from 5 °C to 90 °C under an extra nitrogen pressure of 3 atm at a heating rate of 1 °C/min. Samples were heated twice in order to determine the reversibility of the process. Raw DSC curves were corrected for the instrumental buffer-buffer baseline and normalized by strand concentration to obtain molar heat capacity curves Cp(T). Excess molar heat capacities curves (Cp_exc_) were obtained from Cp(T), by subtracting a baseline obtained by a fourth-order polynomial fit of the pre- and post-transition Cp trends as described elsewhere^43,44^. The number of DSC components to be adopted in the peak deconvolution procedure was selected in order to minimize fitting errors. Cp_exc_ curves were deconvoluted by the NanoAnalyze software using the Gaussians model. The temperatures (T_m_) and enthalpy (ΔH) of strand melting are defined as the temperature at which the Cp_exc_ curve reaches its maximum value and the area under the Cp_exc_(T) peak, respectively.

### ^1^H-NMR measurements

^1^H-NMR spectra were acquired at 5, 25, 37 and 49 °C either on a Varian Unity Inova 700 MHz spectrometer equipped with an HCN triple resonance cryoprobe or on a Varian Unity INOVA 500 MHz spectrometer equipped with a broadband inverse probe with z-field gradient and processed using the Varian VNMR and iNMR (http://www.inmr.net) software packages. All micro RNA samples were prepared at ~0.3 mM concentration by dissolving 75 nmol of each miRNA in 250 µL of 10 mM PBS buffer at pH 6.8. The spectra were acquired as 16,384 data points with a recycle delay of 1.0 s; data sets were zero-filled to 32,768 points prior to Fourier transformation and apodized with a shifted sine bell squared window function. Water suppression was achieved by including a double pulsed-field gradient spin-echo (DPFGSE) module^45,46^ in the pulse sequence prior to acquisition.

### Nuclease stability assay

Nuclease stability assay was performed in 10% Fetal Bovine Serum (FBS) (Sigma) in Dulbecco’s Modified Eagle Medium (DMEM) (Microgem) without phenol red at 37 °C. We used FBS instead of pure nucleases because the former mimics better the physiological conditions in which miRNAs operate. Indeed FBS contains several endo- and exo-nucleases and not a single nuclease^47–49^. For preparation of pre-treated or control samples, 3.5 nmol of oligonucleotide (ON) were dissolved in 125 µL of FBS or proper buffer, respectively. After 72 h of incubation samples were stored at −80 °C for 5 h, then lyophilized and re-dissolved in 10 µL Milli-Q water and 10 µL of loading buffer (glycerol/TBE 1× –30 mM KCl 1:9). 10 µL of the mixture was used for non-denaturing polyacrylamide gel electrophoresis (PAGE).

### Non-denaturing polyacrylamide gel (PAGE)

Non-denaturing gel electrophoresis was performed using 20% polyacrylamide gel, which was run in 1 × TBE (Tris-Borate-EDTA) buffer supplemented with 30 mM KCl, pH 7.0 for 2 h. All samples were loaded at 175 µM concentration in the same run. Electrophoresis was performed at constant voltage of 120 V. Gel was analyzed by UV shadowing. The picture shown in Fig. 5 was taken from the raw image shown in the supporting information (lane 1 = bromophenol blue).

## Supplementary information

Supplementary Information.
