# Supplementary material for: Endogenous and artificial miRNAs explore a rich variety of conformations: a potential relationship between secondary structure and biological functionality
Source: Sci Rep. 2020 Jan 16;10:453. doi: 10.1038/s41598-019-57289-8 (PMC6965629; doi:10.1038/s41598-019-57289-8)

Supplementary information

# Endogenous and artificial miRNAs explore a rich variety of conformations: a potential relationship between secondary structure and biological functionality

C. M. A. Gangemi,[a] S. Alaimo,[b] A. Pulvirenti,[b] Sara García-Viñuales,[c] D. Milardi,[c] A. P. Falanga,[d] M. E. Fragalà,[a] G. Oliviero,[d] G. Piccialli,[e] N. Borbone,[e] A. Ferro,[b*] A. D'Urso,[a*] C. M. Croce,[f*] R. Purrello[a*]

[a] *Department of Chemical Science, University of Catania, Viale A. Doria 6, 95125 Catania, Italy;*

[b] *Bioinformatics Unit, Department of Clinical and Experimental Medicine University of Catania, Italy c/o Department of Mathematics and Computer Science, Viale A. Doria 6, 95125 Catania, Italy;*

[c] *Istituto di Cristallografia CNR, Via P. Gaifami 9, 95126, Catania, Italy;*

[d] *Department of Molecular Medicine and Medical Biotechnologies, University of Naples Federico II, Via Pansini 5, 80131 Napoli, Italy;*

[e] *Department of Pharmacy, University of Naples Federico II, D. Montesano 49, 80131 Napoli, Italy*

[f] *Department of Molecular Virology, Immunology and Medical Genetics, Comprehensive Cancer Center, The Ohio State University, Columbus, OH, 43210 USA.*

**Table of Contents:**

**Experimental Procedures** .................... 2

**Figure S1 – CD spectra of a-miRNA sequences** .................... 4

**Figure S2 – CD melting experiment of a-miR-98 and a-miR-196 sequences** .................... 4

**Table S1 – Parameters relative of thermal transitions of a-miR-23 and a-miR-141 sequences** .................... 5

**Figure S3 – Plot of DSC transitions of a-miR-23 at different concentrations** .................... 5

**Figure S4 – DSC curves of a second heating scan of a-miR-23 at different concentrations** .................... 6

**Figure S5 – Plot of DSC transitions of a-miR-141 at different concentrations** .................... 7

**Figure S6 – DSC curves of a second heating scan of a-miR-141 at different concentrations** .................... 7

**Figure S7 – 1H-NMR spectra of a-miR-141 in PBS recorded at 5 °C and 25 °C.** .................... 8

**Table S2 – Parameters relative of thermal transitions of miR-15a and miR-15b sequences** .................... 9

**Figure S8 – Plot of DSC transitions of miR-15a at different concentrations** .................... 9

**Figure S9 – DSC curves of a second heating scan of miR-15a at different concentrations** .................... 10

**Figure S10 – Plot of DSC transitions of miR-15b at different concentrations** .................... 11

**Figure S11 – DSC curves of a second heating scan of miR-15b at different concentrations** .................... 11

**Figure S12. PAGE of miR-15a and miR-15b** .................... 12

## EXPERIMENTAL PROCEDURES

All the artificial miRNAs (a-miRs) and human miRNAs used in our work were purchased from Integrated DNA Technologies IDT® and used without further purification. Each solid was dissolved in ultra-pure water obtained by Elga Purelab Flex system by Veolia with purity of 18.2 MΩcm, achieving stock solutions with concentration of ~100 µM. Then, by dilution in PBS buffer 10 mM ([KCl] 2.7 mM; [NaCl] 137 mM; pH 6.8) we prepared work and sample solutions. Concentration of a-miRs solutions were checked by UV-Vis measurements using the extinction coefficient for each sequence given by IDT: a-miR-23 $\varepsilon_{260\,nm}$ = 181,600 L/(mol·cm); a-miR-141 $\varepsilon_{260\,nm}$ = 211,700 L/(mol·cm); a-miR-98 $\varepsilon_{260\,nm}$ = 194,200 L/(mol·cm); a-miR-196 $\varepsilon_{260\,nm}$ = 202,200 L/(mol·cm); miR-15a $\varepsilon_{260\,nm}$ = 209,900 L/(mol·cm); miR-15b $\varepsilon_{260\,nm}$ = 211,000 L/(mol·cm).

The sequences of miRNAs used in this study are the following (in *italics* we show the seed region and in **bold** the differences between miR-15a and miR-15b):

a-miR-23: 5'-*UCA AUU UCG* GUC CCG AGU UCC A-3';

a-miR-141: 5'-*UUC CAA UUC* GAG GGG AGG UGG G-3';

a-miR-98: 5'-*UUU CUU AAG* CAC GCC GUU GGG G-3';

a-miR-196: 5'-*UGA GUU UCU* CAG CGA CGG ACC G-3';

miR-15a: 5'-*UAG CAG CAC* AU**A** AUG GUU U**GU G**-3';

miR-15b: 5'-*UAG CAG CAC* AU**C** AUG GUU U**AC A**-3'

By using the "oligoanalyzer tool" provided by IDT® we calculated the conceivable secondary structures likely adopted by each sequence.

### Electronic Circular Dichroism and melting experiments

miRNA samples were analyzed at either 1.4 or 2.8 µM single strand concentration. ECD spectra were recorded at 37°C using a Jasco J-710 spectropolarimeter equipped with a single position Peltier temperature control system. A quartz cuvette with a 1 cm path length was used for all ECD experiments. Conditions were as follows: scanning rate 50 nm/min, data pitch 0.5 nm, digital integration time (D.I.T) 2s, band width 2.0 nm. Each ECD spectrum was an average of at least five scans. The ECD melting experiments were performed within the temperature range 5–90°C using a temperature heating rate of 1°C/min, monitoring the intensity of the miRNA ECD signal at 260 nm.

### Differential Scanning Calorimetry (DSC)

Differential Scanning Calorimetry (DSC) experiments were carried out using a NanoDSC instrument (TA Instruments). Samples were analyzed at total strand concentrations of 18 mg/mL, 24 mg/mL and 40 mg/mL
(i.e. 25 µM, 35 µM and 55 µM, respectively) in a 10 mM PBS buffer pH 6.8. Each sample was heated from 5 °C to 90 °C under an extra nitrogen pressure of 3 atm at a heating rate of 1 °C/min. Samples were heated twice in order to determine the reversibility of the process. Raw DSC curves were corrected for the instrumental buffer-buffer baseline and normalized by strand concentration to obtain molar heat capacity curves Cp(T). Excess molar heat capacities curves ($Cp_{exc}$) were obtained from Cp(T), by subtracting a baseline obtained by a fourth-order polynomial fit of the pre- and post-transition Cp trends as described elsewhere. The number of DSC components to be adopted in the peak deconvolution procedure was selected in order to minimize fitting errors. $Cp_{exc}$ curves were deconvoluted by the NanoAnalyze software using the Gaussians model. The temperatures ($T_m$) and enthalpy ($\Delta H$) of strand melting are defined as the temperature at which the $Cp_{exc}$ curve reaches its maximum value and the area under the $Cp_{exc}(T)$ peak, respectively.

## $^{1}$H-NMR measurements

$^{1}$H-NMR spectra were acquired at 5, 25, 37 and 49 °C either on a Varian Unity Inova 700 MHz spectrometer equipped with an HCN triple resonance cryoprobe or on a Varian Unity INOVA 500 MHz spectrometer equipped with a broadband inverse probe with z-field gradient and processed using the Varian VNMR and iNMR (http://www.inmr.net) software packages. All micro RNA samples were prepared at ~0.3 mM concentration by dissolving 75 nmol of each miRNA in 250 µL of 10 mM PBS buffer at pH 6.8. The spectra were acquired as 16,384 data points with a recycle delay of 1.0 s; data sets were zero-filled to 32,768 points prior to Fourier transformation and apodized with a shifted sine bell squared window function. Water suppression was achieved by including a double pulsed-field gradient spin-echo (DPFGSE) module [46, 47] in the pulse sequence prior to acquisition.

## Nuclease stability assay

Nuclease stability assay was performed in 10% Fetal Bovine Serum (FBS) (Sigma) in Dulbecco's Modified Eagle Medium (DMEM) (Microgem) without phenol red at 37°C. We used FBS instead of pure nucleases because the former mimics better the physiological conditions in which miRNAs operate. Indeed FBS contains several endo- and exo-nucleases and not a single nuclease (*Hahn, J., Wickham, S. F. J., Shih, W. M. & Perrault, S. D. Addressing the instability of DNA nanostructures in tissue culture. ACS Nano* ***8****, 8765–8775, 2014; Virgilio, A. et al. Monomolecular G-quadruplex structures with inversion of polarity sites: New topologies and potentiality. Nucleic Acids Res.* ***45****, 8156–8166, 2017; Conway, J. W., McLaughlin, C. K., Castor, K. J. & Sleiman, H. DNA nanostructure serum stability: Greater than the sum of its parts. Chem. Commun.* ***49****, 1172–1174, 2013)*. For preparation of pre-treated or control samples, 3.5 nmol of oligonucleotide (ON) were dissolved in 125 µL of FBS or proper buffer, respectively. After 72 h of incubation samples were stored at –80 °C for 5 h, then lyophilized and re-dissolved in 10 µL Milli-Q water and 10 µL of loading buffer (glycerol/TBE 1× –30 mM KCl 1:9). 10 µL of the mixture was used for non-denaturing polyacrylamide gel electrophoresis (PAGE).

## Non-denaturing polyacrylamide gel (PAGE)

Non-denaturing gel electrophoresis was performed using 20% polyacrylamide gel, which was run in 1×TBE (Tris-Borate-EDTA) buffer supplemented with 30 mM KCl, pH 7.0 for 2 h. All samples were loaded at 175 µM concentration in the same run. Electrophoresis was performed at constant voltage of 120 V. Gel was analyzed by UV shadowing. The picture shown in Figure 5 was taken from the raw image shown below (lane 1 = bromophenol blue).

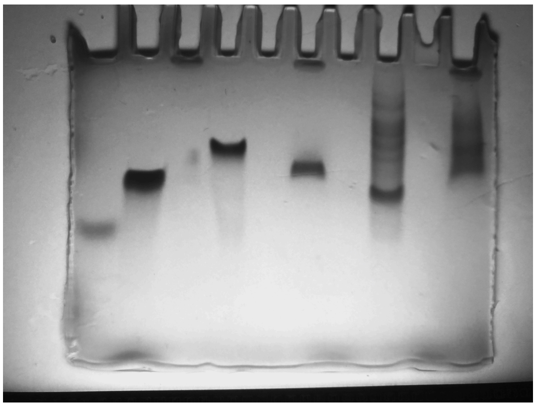

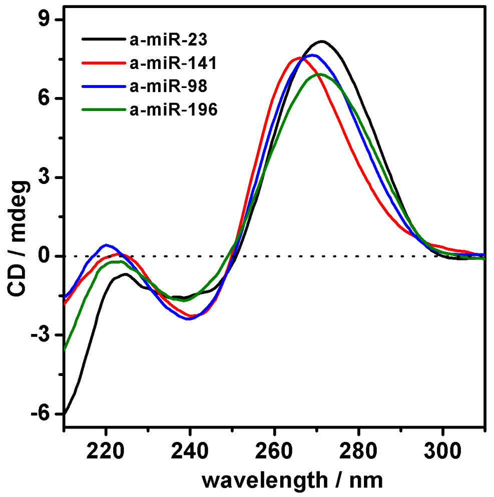


**Figure S1.** CD-spectra of a-miR-23 (black curve), a-miR-141 (red curve), a-miR-98 (blue curve), a-miR-196 (green curve), 1.4 μM in PBS 10 mM, pH = 6.8, 37°C.

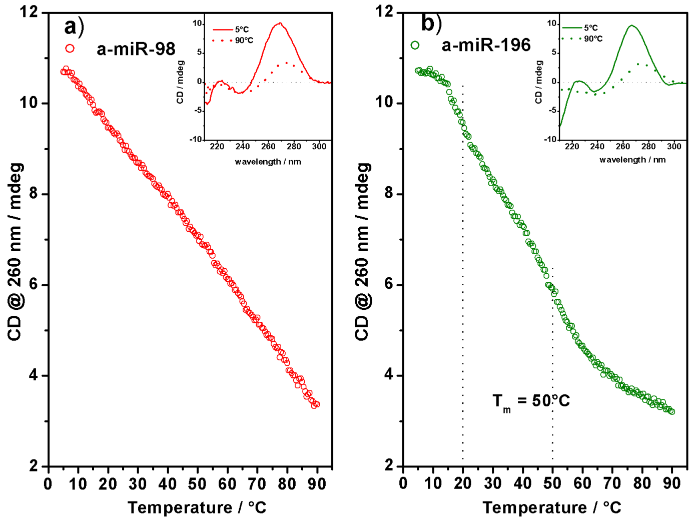


**Figure S2.** CD-melting curves of a) a-miR-98; b) a-miR-196; in PBS 10 mM, pH = 6.8. Insets show the CD spectra of a-miR sequences at 5°C (solid curves) and 90°C (dotted curves)

**Table S1**. Parameters relative to the two components of thermal transitions of a-miR-23 and a-miR-141 sequences.

| | | **amiR-23** | | | | **amiR-141** | | | |
|---|---|---|---|---|---|---|---|---|---|
| | | **Peak 1** | | **Peak 2** | | **Peak 1** | | **Peak 2** | |
| | **Conc (mg/mL)** | **Tm$_1$ (°C)** | **ΔH$_1$ (kJ/mol)** | **Tm$_2$ (°C)** | **ΔH$_2$ (kJ/mol)** | **Tm$_1$ (°C)** | **ΔH$_1$ (kJ/mol)** | **Tm$_2$ (°C)** | **ΔH$_2$ (kJ/mol)** |
| ***Heating 1*** | ***0.18*** | 42.1 ± 0.5 | 40.2 ± 2.3 | 46.6 ± 0.7 | 22.5 ± 2.9 | 60.6 ± 0.2 | 63.1 ± 3.1 | 73.1 ± 0.5 | 29.0 ± 7.2 |
| | ***0.24*** | 41.9 ± 0.6 | 48.5 ± 3.0 | 47.1 ± 0.7 | 19.0 ± 2.7 | 60.1 ± 0.5 | 81.4 ± 4.1 | 72.8 ± 0.6 | 31.5 ± 6.2 |
| | ***0.40*** | 44.5 ± 0.5 | 80.1 ± 6.7 | 46.8 ± 0.6 | 18.7 ± 1.7 | 61.2 ± 0.8 | 101 ± 7.0 | 73.9 ± 0.7 | 31.7 ± 5.9 |
| ***Heating 2*** | ***0.18*** | 43.5 ± 0.3 | 58.0 ± 4.0 | 47.1 ± 0.9 | 9.0 ± 2.0 | 50.1 ± 0.4 | 63.3 ± 7.0 | 64.3 ± 0.6 | 21.2±0.6 |
| | ***0.24*** | 43.4 ± 0.5 | 51.7 ± 3.6 | 48.0 ± 0.1 | 11.5±0.3 | 53.5 ± 0.7 | 76.4 ± 5.9 | 67.2 ± 0.5 | 12.7±0.8 |
| | ***0.40*** | 45.1 ± 0.6 | 59.0 ± 3.9 | 47.2 ± 0.9 | 11.9±20.8 | 55.4 ± 0.1 | 92.7 ± 6.2 | 68.5 ± 0.7 | 7.2±0.1 |

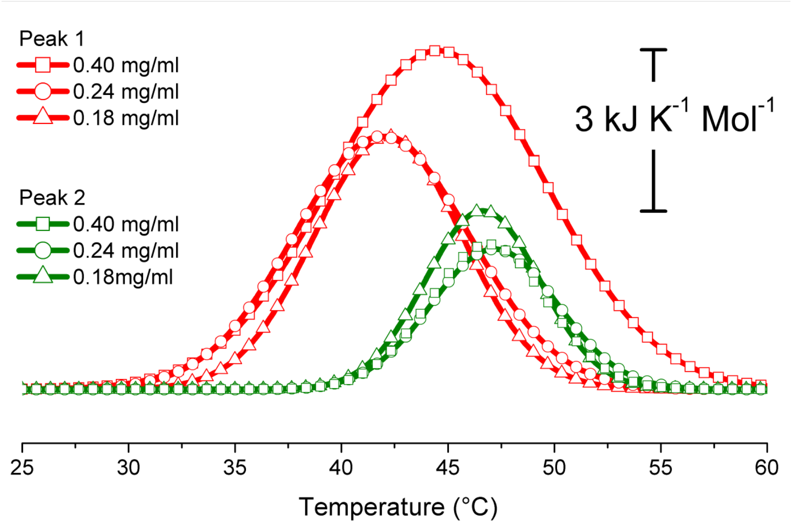


**Figure S3.** Plot of the 1st (red) and 2nd (green) component of a-miR-23 DSC transitions at different concentration (0.18, 0.24 and 0.40 mg/mL) in 10 mM PBS, 100 mM NaCl at pH 6.8.

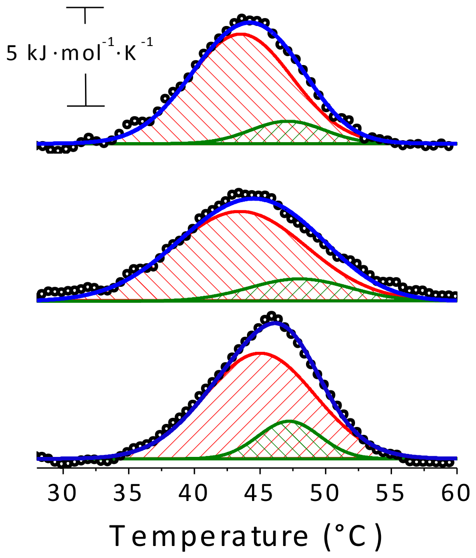


**Figure S4.** DSC curves of a second heating scan of a-miR-23 at different concentrations (up 0.18 mg/mL, middle 0.24 mg/mL and down 0.40 mg/mL) in 10 mM PBS, 100 mM NaCl at pH 6.8. DSC curves (black open circles) are deconvoluted in two components: the red and green curves correspond to the 1st and 2nd transition, of a biphasic melting curve. The sum of the two components is reported as a blue line.

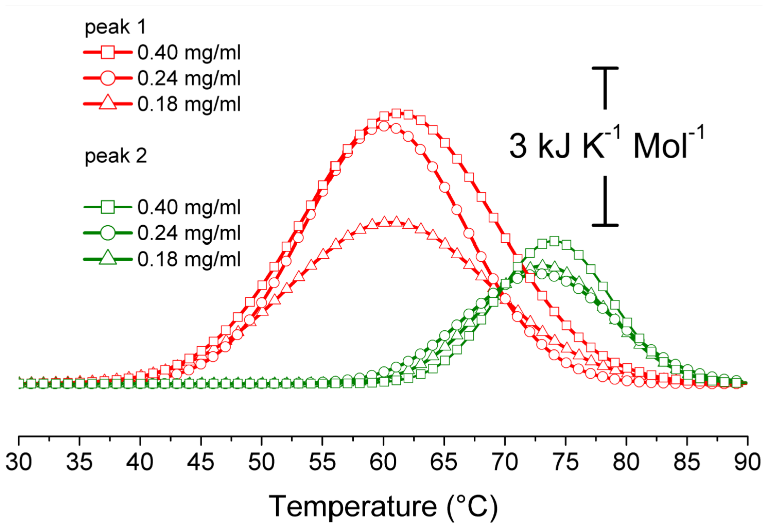


**Figure S5.** Plot of the 1st (red) and 2nd (green) component of a-miR-141 DSC transitions at different concentration (0.18, 0.24 and 0.40 mg/mL) in 10 mM PBS, 100 mM NaCl at pH 6.8.

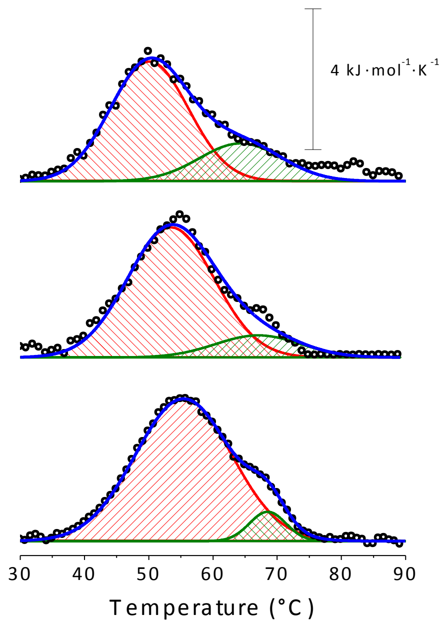


**Figure S6.** DSC curves of a second heating scan of a-miR-141 at different concentrations (up 0.18 mg/mL, middle 0.24 mg/mL and down 0.40 mg/mL) in 10 mM PBS, 100 mM NaCl at pH 6.8. DSC curves (black open circles) are deconvoluted in two components: the red and green curves correspond to the 1st and 2nd transition, of a biphasic melting curve. The sum of the two components is reported as a blue line.

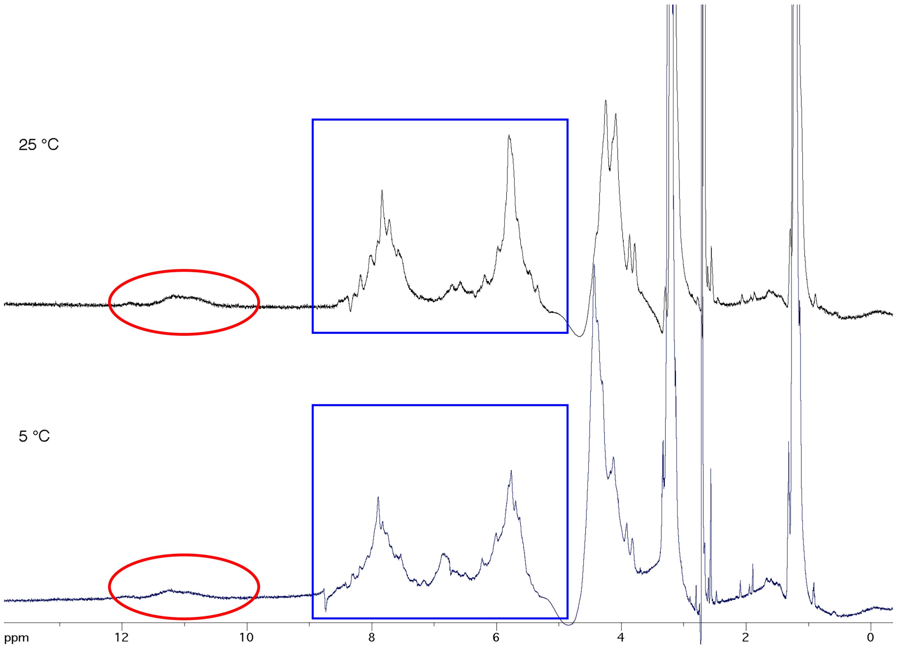


**Figure S7**. 1H-NMR spectra of a-miR-141 in PBS recorded at 5 °C and 25 °C. The blue box highlights the anomeric and aromatic protons. The red oval highlights the H-bonded imino protons.

**Table S2**. Parameters relative to the two components of thermal transitions of a-miR-15-a and a-miR-15-b sequences.

| | | miR-15a | | | | miR-15b | | | |
|---|---|---|---|---|---|---|---|---|---|
| | | Peak 1 | | Peak 2 | | Peak 1 | | Peak 2 | |
| | Conc (mg/mL) | $T_1$ (°C) | $\Delta H_1$ (kJ/mol) | $T_2$ (°C) | $\Delta H_2$ (kJ/mol) | $T_1$ (°C) | $\Delta H_1$ (kJ/mol) | $T_2$ (°C) | $\Delta H_2$ (kJ/mol) |
| *Heating 1* | ***0.18*** | 32.0 ± 0.6 | 28.1 ± 2.1 | 45.3 ± 0.4 | 83.4 ± 6.0 | 21.2 ± 0.6 | 29.0 ± 2.7 | 27.7 ± 0.7 | 136.1 ± 10.1 |
| | ***0.24*** | 30.7 ± 0.7 | 10.1 ± 3.0 | 45.4 ± 0.3 | 116.4 ± 9.6 | 18.3 ± 0.2 | 14.1 ± 2.7 | 27.5 ± 0.6 | 153.4 ± 12.2 |
| | ***0.40*** | 31.9 ± 0.7 | 11.0 ± 2.9 | 46.0 ± 0.2 | 112.1 ± 10.1 | 19.9 ± 0.8 | 6.7 ± 1.7 | 28.7 ± 0.3 | 162.8 ± 8.0 |
| *Heating 2* | ***0.18*** | 37.09±0.05 | 3.6±0.3 | 45.65±0.04 | 48.8±0.3 | 20.9 ± 0.9 | 28.0 ± 3.1 | 27.0 ± 0.7 | 136 ± 13.1 |
| | ***0.24*** | - | - | 46.74±0.02 | 44.5±0.3 | 18.6 ± 0.6 | 14.7 ± 2.9 | 26.9 ± 0.4 | 151.7 ± 12.9 |
| | ***0.40*** | - | - | 46.33±0.01 | 43.7±0.2 | 20.2 ± 0.8 | 14.0 ± 3.1 | 28.5 ± 0.5 | 157 ± 10.1 |

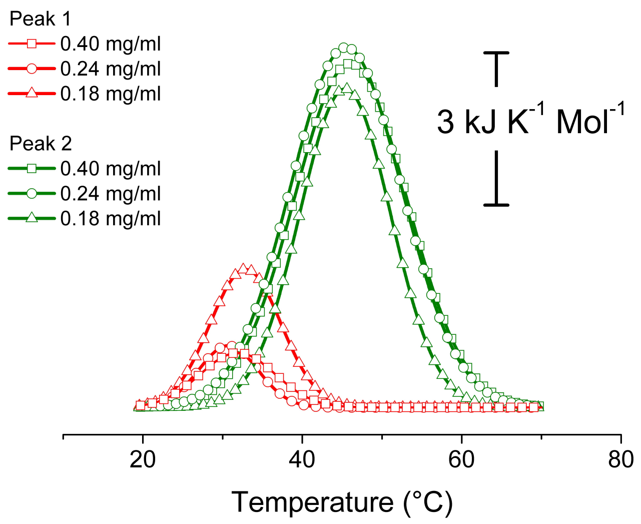


**Figure S8.** Plot of the 1st (red) and 2nd (green) component of a-miR-15-a DSC transitions at different concentration (0.18, 0.24 and 0.40 mg/mL) in 10 mM PBS, 100 mM NaCl at pH 6.8.

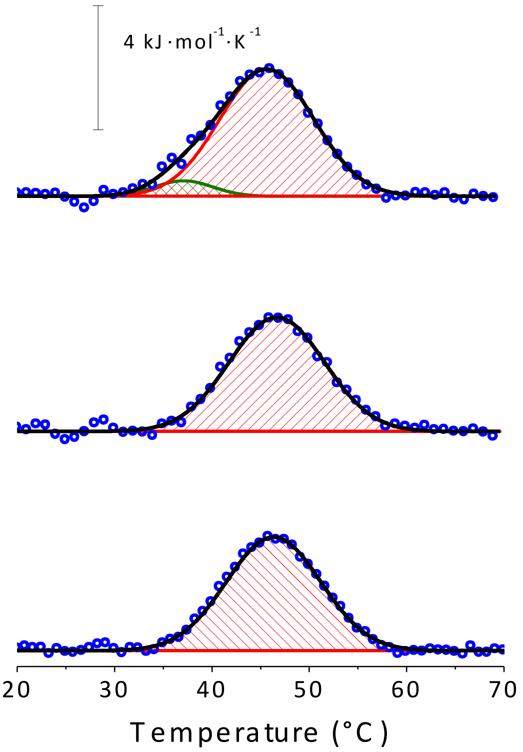


**Figure S9.** DSC curves of a second heating scan of a-miR-15a at different concentrations (up 0.18 mg/mL, middle 0.24 mg/mL and down 0.40 mg/ml) in 10 mM PBS, 100 mM NaCl at pH 6.8. DSC curves (black open circles) are deconvoluted in two components: the red and green curves correspond to the 1st and 2nd transition, of a biphasic melting curve. The sum of the two components is reported as a blue line.

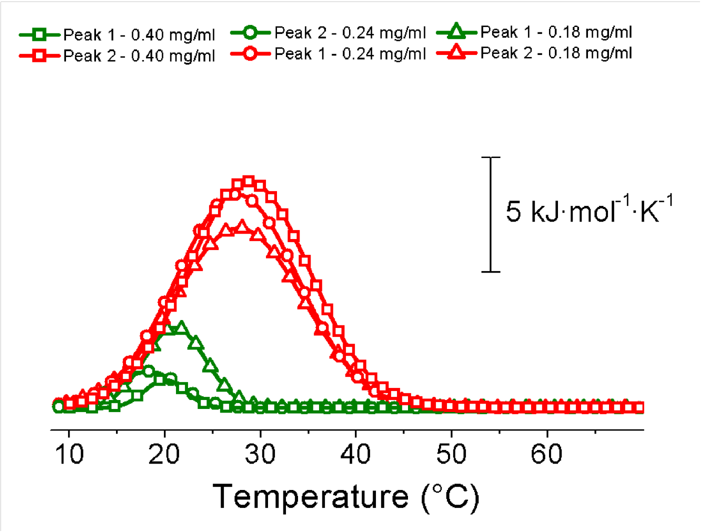


**Figure S10.** Plot of the 1st (red) and 2nd (green) component of a-miR-15b DSC transitions at different concentrations (0.18, 0.24 and 0.40 mg/mL) in 10 mM PBS, 100 mM NaCl at pH 6.8.

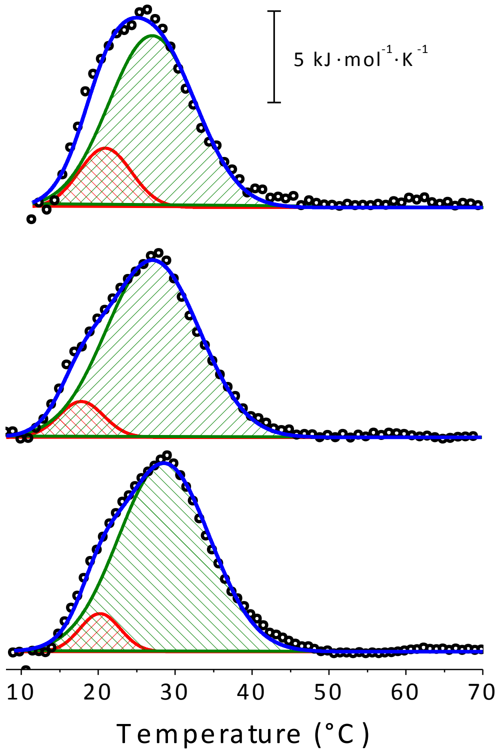


**Figure S11.** DSC curves of a second heating scan of a-miR-15b at different concentrations (up 0.18 mg/mL, middle 0.24 mg/mL and down 0.40 mg/mL) in 10 mM PBS, 100 mM NaCl at pH 6.8. DSC curves (black open circles) are deconvoluted in two components: the red and green curves correspond to the 1st and 2nd transition, of a biphasic melting curve. The sum of the two components is reported as a blue line.

In the absence of FBS, miR-15a and miR-15b migrated mostly as single bands, with the band of miR-15a (Figure S10, lane 3) migrating slightly faster than that of miR-15b (Figure S10, lane 5), in agreement with the spectroscopic data that indicated the formation of distinct secondary structures for the two endogenous miRNAs. When we loaded the samples which were incubated in the FBS-containing buffer for 72 h at 37 °C, we obtained PAGE profiles characterized each by the presence of two less intense and smeared bands (Figure S10, lanes 4 and 6) with electrophoretic mobility equal or lower than that of the corresponding band observed in lanes 3 and 5, but we did not see any faster band attributable to nucleases-induced degradation products of miR-15a and miR-15b."

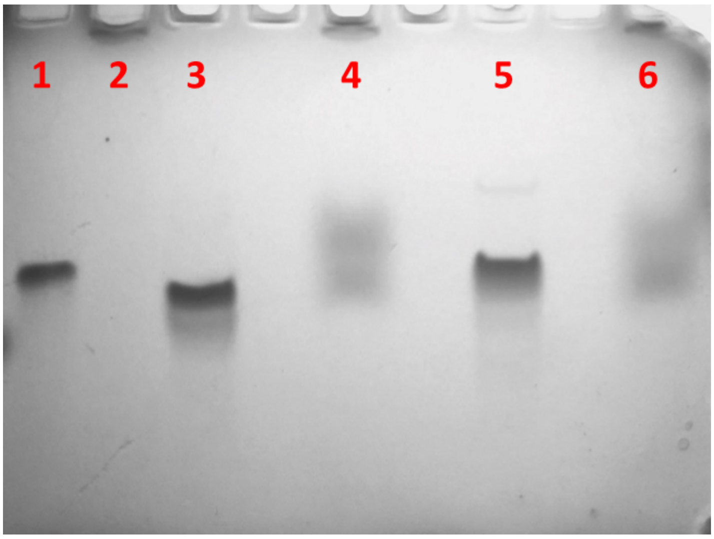


**Figure S12.** PAGE of miR-15a and miR-15b performed 72 h after incubation at 37 °C with DMEM buffer (lanes 3 and 5, respectively) or DMEM + 10% FBS (lanes 4 and 6, respectively). The PAGE behavior of the control T24 RNA sequence in the absence or presence of FBS nucleases is shown in lanes 1 and 2, respectively. This figure was taken from the raw image shown below.

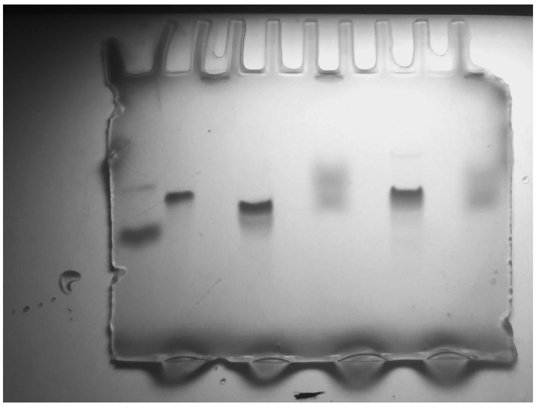

Supplement: Supplementary file 1 — Supplementary Information. [file 41598_2019_57289_MOESM1_ESM.pdf]
